# An increase in p62/NBR1 levels in melioidosis patients of Sri Lanka exhibit a characteristic of potential host biomarker

**DOI:** 10.1099/jmm.0.001242

**Published:** 2020-08-20

**Authors:** Kamal U. Saikh, Cyra M. Ranji, Robert G. Ulrich, Enoka Corea, Aruna Dharshan De Silva, Mohan Natesan

**Affiliations:** ^1^​ Department of Immunology, United States Army Medical Research Institute of Infectious Diseases, Frederick, MD, USA; ^2^​ Department of Microbiology, University of Colombo, Colombo, Sri Lanka; ^3^​ Division of Vaccine Discovery, La Jolla Institute of Allergy and Immunology, La Jolla, CA, USA; ^4^​ Department of Paraclinical Sciences, Faculty of Medicine, Kotelawala Defence University, Ratmalana, Sri Lanka

**Keywords:** melioidosis, *Burkholderia pseudomallei*, autophagy, p62, NBR1, cytokines

## Abstract

**Introduction:**

Melioidosis, caused by *Burkholderia pseudomallei *, in endemic areas, poses a challenge for treating the diseased populations without accurate diagnosis, and the disease-specific biomarkers linked with the infection have yet to be reported. Due to the invasive nature of the causative agent, *Burkholderia pseudomallei *, host innate effector mechanisms, including autophagy are known to be activated, resulting in differential expression of cellular proteins and immune markers. Identification of a disease-specific biomarker associated with *B. pseudomallei* infection will be helpful to facilitate rapid confirmation of melioidosis, which would enable early treatment and therapeutic success.

**Aim:**

We aimed to assess the levels of a host autophagy component, p62/NBR1, which function as a cargo-receptor in the process of autophagy activation leading to the degradation of ubiquitin-coated intracellular bacteria in which p62/NBR1 itself is degraded in the clearance of the pathogen. We further probed the extent of intracellular p62/NBR1 degradation and assessed its potential as a melioidosis biomarker.

**Methodology:**

We analysed peripheral blood mononuclear cell (PBMC) lysates using an ELISA-based assay for detecting cytosolic autophagy-related proteins p62/NBR1. We measured p62/NBR1 levels in diseased (confirmed *B. pseudomallei* infection) and non -diseased populations and utilized receiver operating characteristic (ROC) curve and max Youden index analysis for evaluating potential disease biomarker characteristics.

**Results:**

Our results revealed a three to fivefold increase in p62/NBR1 levels confirmed melioidosis cases compared to uninfected healthy donors. Comparable to p62/NBR1, levels of cytosolic LC3-I levels also increased, whereas the levels of degraded membrane bound form LC3-II was low, suggesting autophagy deficiency. Proinflammatory serum cytokine response, particularly IL-6, was consistently higher alongside *B. pseudomallei* infection in comparison to healthy controls.

**Conclusions:**

ROC curve and max Youden index analysis suggest that increased p62/NBR1 levels in diseased populations display characteristics of a potential disease biomarker in melioidosis and illustrates that an elevated p62/NBR1 level, in conjunction with *B. pseudomallei* infection associated with autophagy deficiency.

## Introduction

Melioidosis is a serious invasive disease, caused by a Gram-negative bacterial pathogen *B. pseudomallei *, which is endemic in Southeast Asia and Northern Australia. It is an environmental pathogen found in soil and water, and infections are commonly contracted through contact with contaminated soil or water [1]. The pathogen can be acquired by humans via inhalation, inoculation or ingestion, with the largest number of infections developing amid the rainy season [2]. *B. pseudomallei* after gaining entry into cytosol, survives and replicates in infected cells. It is resistant to numerous antibiotics, and, if not treated, the mortality rate varies from 14 % in northern Australia to 43 % in northeast Thailand to as high as 61.5 % in Cambodia [3–5], and 20 % in Sri Lanka [6]. The clinical presentations of melioidosis vary with manifestations that range from localized abscess formation to acute pneumonia and overwhelming septicemia [2, 7]. Melioidosis can affect all age groups. While acute cases commonly present within 1–21 days after infection, the symptoms are usually less severe in chronic cases but persist for weeks or months [2, 3]. Diagnosis of melioidosis can be due to varied clinical manifestations. Further complicating matters, *B. pesudomallei* has been designated as a category B agent by the US Centers for Disease Control (CDC) restricting work to select agent compliant biosafety level 3 containment facilities [8, 9].

*B. pseudomallei* generate a variety of clinical manifestations, depending on the tissue infected, and can maintain a survival advantage in infected hosts as well as the environment [10]. Infection initially occurs in epithelial cells of the mucosal surface or broken skin, and then spreads to various cell types including phagocytic and non-phagocytic cells [11, 12]. *B. pseudomallei* following endocytosis, can be seen in endocytic vesicles and later within cytoplasm where it replicates [13–17]. Thus, *B. pseudomallei* can multiply within phagocytes (including neutrophils, monocytes and macrophages) without activating a bactericidal response [11, 12]. While lysosome fusions are detected within *B. pseudomallei-*infected macrophages, which suggest that limited degradation of the pathogens can occur, however proliferation of surviving bacteria ultimately overwhelms. Reports also indicate that dormant or in-apparent infections likely occur and may recrudesce in severe fulminant form months and even years after exposure [18]. Reoccurence, occurs in approximately 9 % of patients [3]. Recurrent, relapsed and reinfection of melioidosis patients in endemic areas suggest lack of complete clearance of the pathogen, therefore, posing significant public health concerns. Further, *B. pseudomallei* can remain latent for an extended period ranging from 19 to 29 years before immunosuppression or other host stress responses reactivate bacterial proliferation and subsequently melioidosis develops [18–21]. This indicates that *B. peudomallei* evades immune surveillance by entering a dormant state [21]. *B. pseudomallei* may trigger partial autophagy, an innate effector mechanism by a type-three secretion system (T3SS)-dependent process in which T3SS most likely plays an essential role in the evasion of autophagy. However, the possible mechanisms by which *B. pseudomallei* remains undetected is still unknown. Thus, accurate diagnoses of melioidosis can be difficult due to its wide range of clinical manifestations. Hence tedious, time-consuming laboratory diagnostic tests are required for the presence of the pathogen and disease confirmation. Identification and testing of a specific biomarker confirmed with *B. pseudomallei* infected diseased population at point of care would be very useful for early diagnosis of infection, and treatment, given that diagnostic biomarkers for melioidosis are recognized.

Autophagy is a known conserved degradative and recycling pathway that plays a role in various biological processes, including the cellular response to starvation [22] and host-defense through degradation of invading bacteria, promoting cell survival [23–25]. Both p62 and NBR1 act as cargo receptors for selective autophagosomal degradation of ubiquitylated targets, thus, autophagy is responsible for the degradation of p62/NBR1. Under normal conditions, basal autophagy continuously clears p62/NBR1 and associated cargo from the cytoplasm, however under conditions of autophagy deficiency, p62/NBR1 and associated cargo accumulates in the cytoplasm. Therefore, studies correlating host analysis of cellular responses with *B. pseudomallei* infection particularly activation of the host immune effector mechanism during melioidosis and septicemic conditions caused by other pathogens, could potentially identify differentially expressed immune markers. Identification of such specific host-factor(s) modulation linked to intracellular *B. pseudomallei* infection may serve as a potential biomarker, and would be immensely helpful for treating the disease and eradication of the pathogen [26]. While the current understanding of the autophagy process directed against pathogens has been studied extensively, selective autophagy, including *B. pseudomallei* infection *in vivo*, and intracellular persistence of this bacteria has not been fully elucidated, in particular as it pertains to corroboration of the disease. Also it remains unclear whether or not components of autophagy machinery may reveal other roles, such as projection of infection, in disease confirmation or disease-associated differentially expressed cellular components including p62/NBR1 has not been explored. Our earlier study in a mouse model of melioidosis suggest that the persistence of *B. pseudomallei* is associated with autophagy deficiency where the autophagy component p62/NBR1 was significantly up regulated [27]. In this study, we aimed to extend this observation in human disease by analysing samples from confirmed melioidosis patients and evaluated whether detectable levels of p62/NBR1 in infected mononuclear cells may be significantly higher than in normal healthy controls, which could be utilized as a potential biomarker of melioidosis.

## Results

### Patient enrolment

Patient enrolment and clinical characteristics of the study population included here were partly reported elsewhere [28]. Clinical samples were collected over a period of 2 years (2013–2015). In this study, we analysed 295 samples that were placed into seven different categories as shown in Table 1. Among 295 samples, 116 were confirmed cases of melioidosis (* B. pseudomallei* culture positive). Out of the 116 confirmed cases of melioidosis, 27 were classified as convalescent (blood collected from the patient while undergoing antibiotic treatment). Among the melioidosis-negative patient samples, 28 characterized as sepsis and 38 samples were considered as leptospirosis based on clinical symptoms. In total, 46 patients were probable cases of melioidosis as a result of high antibody titre but were culture negative. All these samples were analysed by measuring p62/NBR1 levels in PBMCs. Among 295 samples, 143 samples belonging to the seven categories were analysed for proinflammatory cytokines (TNF-α, IL-1β, IFN-γ and IL-6) in serum.

**Table 1.** Clinical characteristics and number of study population

Characteristics	No. of patients
Healthy control	42
Melioidosis-negative suspected leptospirosis	34
Melioidosis-negative sepsis	28
Melioidosis probable	46
Melioidosis confirmed	116
Melioidosis relapsed	2
Melioidosis convalescent	27

### p62/NBR1 levels were increased in PBMCs of melioidosis patients

**Fig. 1.**
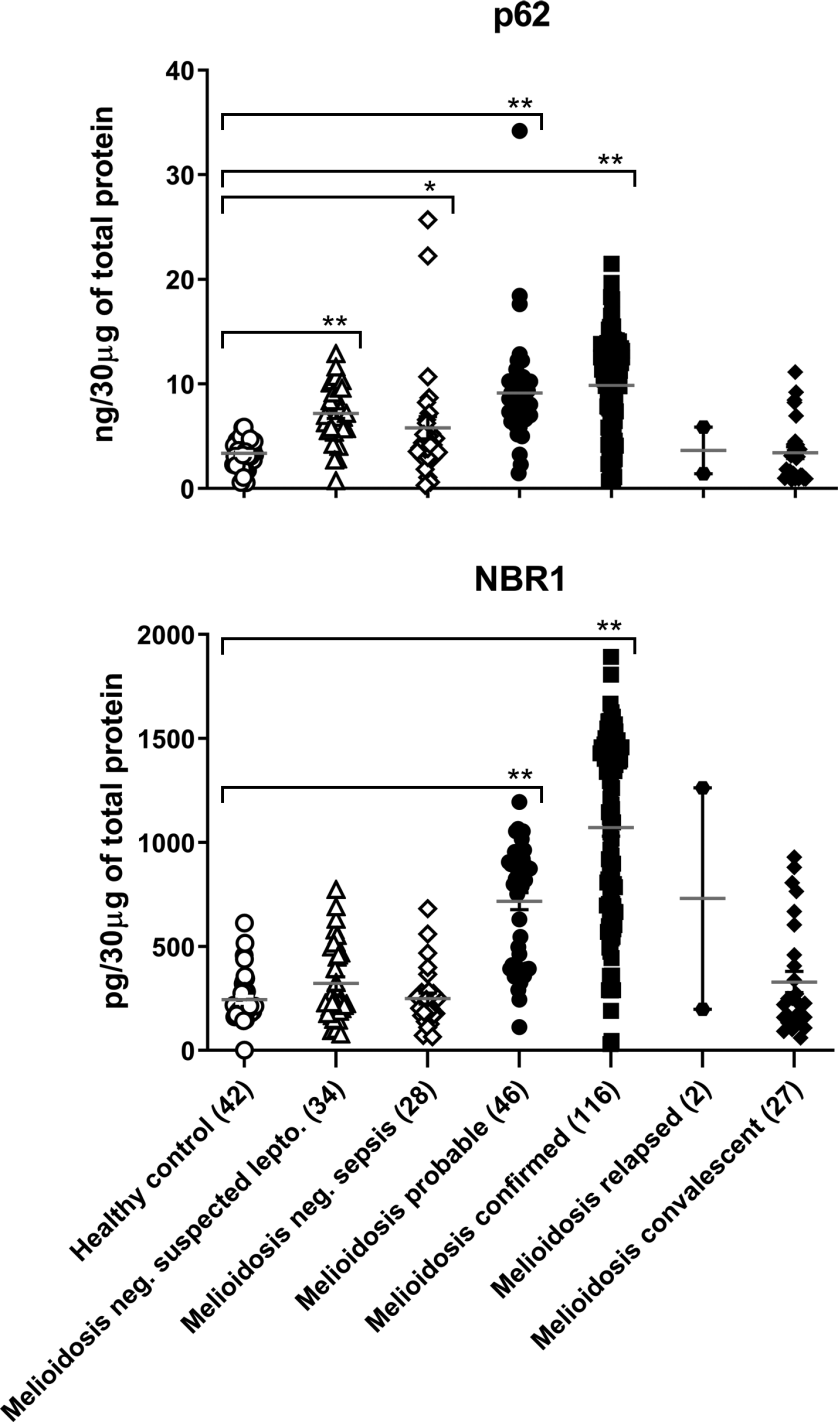
Increase in cytosolic p62 and NBR1 levels in PBMCs of melioidosis patients. Total (5×10^6^ cells) purified PBMCs were lysed as described in Methods. Overall, 30 µg of lysates were used to measure p62 and NBR1 as described elsewhere [27]. Data presented as p62 ng/30 µg of total protein and NBR1 pg/30 µg of total protein, ** represent *P*-value ≤0.0001 and * *P*-value ≤0.05 with post-hoc pairwise comparisons with healthy controls using Dunn’s test, and complete statistical data analysis are also shown in Table S1.

Activation of autophagy is a cell intrinsic innate immune effector mechanism that eliminates infectious agents that access the cytosol. In the autophagy activation process, p62 and NBR1, which link ubiquitinated targets, act as receptors for selective autophagosomal degradation of targets in which p62 and NBR1 are also degraded. We examined intracellular p62/NBR1 levels, in lysates of purified PBMCs from 295 samples, as shown in Table 1, and utilized an ELISA assay to measure cytosolic levels of p62 and NBR1 in confirmed melioidosis patients with *B. pseudomallei* infection, probable cases of melioidosis with high antibody titre specific to *B. pseudomallei* but were culture negative, healthy controls as well as *B. pseudomallei *-negative with suspected cases of sepsis, and *B. pseudomallei *-negative suspected cases of leptospirosis. Our results showed a significant (fourfold) increase in p62 levels in confirmed melioidosis patients with *B. pseudomallei* infection compared to healthy controls, *P*-value ≤0.0001 (Fig. 1, Table S1, available in the online version of this article). Also all patients with probable cases of melioidosis had higher levels of p62 compared to healthy controls but lower than confirmed melioidosis patients, *P*-value ≤0.0001 (Fig. 1, Table 2). A small detectable increase in p62, but distinctly lower than confirmed or probable cases of melioidosis samples, was also noted in suspected cases of sepsis, *P*-value ≤0.0582 and *B. pseudomallei *-negative suspected cases of leptospirosis *P*value ≤0.0001. Similar to p62, cytosolic NBR1 had significantly higher levels (fivefold) in confirmed, as well as probable, cases of melioidosis than in healthy controls, or meliodoisis-negative sepsis or meliodoisis-negative suspected leptospirosis categories. Although very low levels of p62 were observed in meliodoisis-negative *B. pseudomallei* sepsis and suspected leptospirosis patients, the NBR1 levels were distinctly statistically significant (Fig. 1, Table S1). Kruskal–Wallis analysis with post-hoc pairwise comparisons using Dunn’s test indicate statistically significant only with probable and confirmed cases of melioidosis compared to healthy controls. The interquartile range (IQR) is an important measure that indicates the amount of variability within a data set divided into quartiles between disease and healthy control. Data analysis showed that the value of IQR for p62 in confirmed and probable cases of melioidosis were 6.75, 12.86, and 6.70, 10.27 respectively compared to healthy controls, which were 2.68, 4.11. Similarly, IQR for NBR1 in confirmed and probable cases of melioidosis were 695.5, 1449.66, 399.64 and 918.5 respectively compared to healthy controls, which were 180.23 and 284.6. (Tables S1 and Table 2). These data analyses of p62 and NBR1 as noted in Fig. 1 revealed that the three to fivefold increase in intracellular accumulation of p62/NBR1 with *B. pseudomallei* infection in melioidosis patients compared to healthy controls display a characteristic in diseased population. Further, the increase in p62/NBR1 with autophagy deficiency suggests that *B. pseudomallei* were not completely cleared out and persist intracellularly. It is important to note that earlier reports indicate that recurrence, due to reactivation of latent infection, is also common with meliodosis [29].

**Table 2.** ROC curve analysis of probability diagnostic point of melioidosis in confirmed melioidosis from healthy control

Analyte	AUC	Analyte	Max Youden index	Probability of melioidosis at diagnositc point	Approximate diagnostic cut-point (concentration units)
IFN-gamma	0.859	IFN-gamma	0.769231	0.71822	6.22
IL-1B	0.828	IL-1B	0.65641	0.729702	0.27
IL-6	0.967	IL-6	0.848718	0.758648	0.70
NBR1	0.963	NBR1	0.855501	0.807458	535.63
TNF-alpha	0.883	TNF-alpha	0.744872	0.656327	1.92
p62	0.892	p62	0.767241	0.787391	6.40

### Serum cytokines TNF-α, IL-1β, IFN-γ and IL-6 response with *B. pseudomallei* infection

**Fig. 2.**
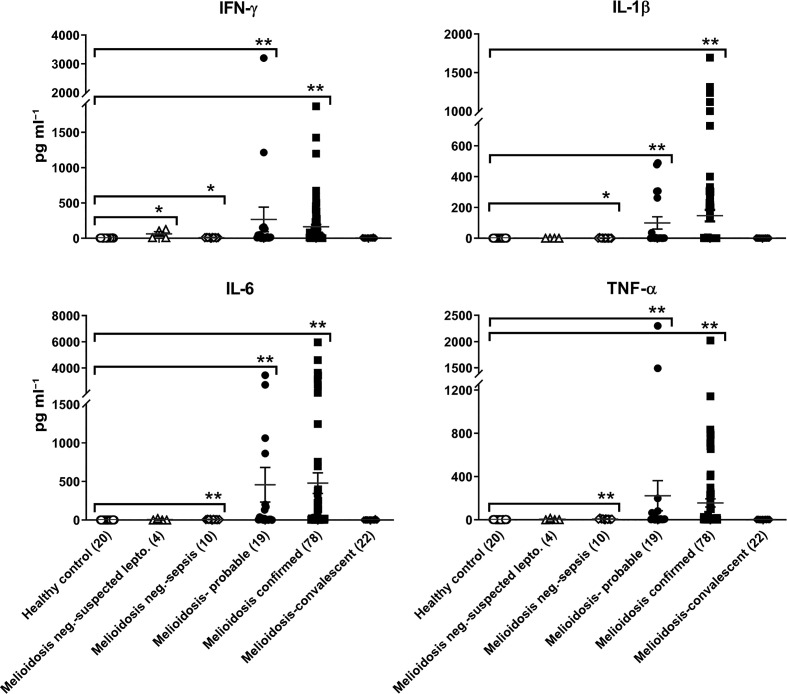
Proinflammatory serum cytokine response in melioidosis. The serum collected from patient blood was used for measuring cytokines by MSD assay as described in Methods. ** represent *P*-value ≤0.0001 and * *P*-value ≤0.05 with post-hoc pairwise comparisons with healthy controls using Dunn’s test, and also complete statistical analysis are shown in Table S1.

A durable, broad, cellular immune response is essential for protection against progression of infection and for bacterial clearance. We analysed proinflammatory cytokine (TNF-α, IL-1β, IFN-γ and IL-6) response in serum of 153 samples of the seven different categories of melioidosis and non-melioidoisis populations as well as healthy controls. Our results showed that while a minor increase in TNF-α, IL-1β and IFN-γ was observed compared to healthy or non-melioidosis patients, a significant increase in IL-6 was evident in confirmed and probable cases of melioidosis patients (Fig. 2, Table S1). These proinflammatory serum cytokine responses were barely detectable in healthy controls (Fig. 2). Among the proinflammatory cytokines, the IQR values for IL-6 were 2.66, 197.62 and 4.49, 202.17 respectively and consistently remained higher in confirmed and probable cases of melioidosis compared to healthy controls, which were 0.30, 0.50 (Table S1). Earlier findings on gene-expression profiles of human cytokine responses in *B. pseudomallei* infection [30, 31], which showed a dominant TH2 and TH17-type-cytokine response, suggest that their dysregulation at initial stages of infection may play an essential role in disease pathogenesis. However, an important point to note is that persistence of IL-6 levels were not detected in convalescent samples including IL-6, further suggesting that dysregulation or deficiency in activation of host strong innate effector mechanism may be involved in lack of clearing the pathogen or persistent as dormant.

### ROC curve analysis of p62/NBR1 and proinflammatory cytokines in melioidosis patients

ROC curve is generally used to evaluate characteristics of a biomarker for classifying disease status in determining the status of a diseased and non-diseased population. In most cases, biomarker levels for a certain disease population are different, usually higher, than in the corresponding non-diseased population. The Youden index (*J*), the maximum potential effectiveness of a biomarker, is a common summary measure of the ROC curve, which is a plot of sensitivity (Se) versus 1-specificity (p) at all possible concentrations is shown in (Table 2). Data set of p62 and NBR1 using ROC curve analysis assuming 0=normal, and, 1=melioidosis showed Youden index 0.767 and 0.8.55 with a probability of melioidosis at diagnostic point 0.787 and 0.807 with an approximate diagnostic cut-point 6.40 ng and 535.63 pg (concentration unit), respectively, per 30 µg of total protein (Table 2, Fig. S1). The concentration range cut-point in the data plot suggests a good sensitivity indicator point of the disease diagnosis of melioidosis with *B. pseudomallei* infection compared to controls. Similar to p62/NBR1, among the cytokines IL-6 appeared to be a good indicator of melioidosis with *B. pseudomallei* infection.

### Intracellular accumulation of p62 and LC3 correlate with autophagy deficiency

When autophagy is induced, the p62 protein is capable of binding to ubiquitylated proteins, as well as autophagosome membrane proteins LC3, to target protein aggregates that access the cytosol including intracellular pathogens for lysosomal degradation and p62 itself is degraded remaining at low levels. The conversion of the cytosolic form of LC3 (LC3-I, 18 kDa) to the autophagosomal-associated form (LC3-II, 16 kDa) is used as a specific indicator for the formation of an autophagosome. To confirm that an increase in accumulation of cytosolic p62 in *B. pseudomallei* infected PBMCs and PBMCs of healthy controls, as well as suspected cases due to autophagy deficiency, which can be correlated with the lack of conversion of LC3-I to LC3-II, we examined p62 and LC3 levels by Western-blot analysis. Our results demonstrated that cell extracts of PBMCs from randomly selected confirmed *B. pseudomallei* infected samples as well as melioidosis-negative sepsis and healthy controls showed strong presence of p62 (60 kDa) protein when probed with anti-p62 antibody (Fig. S2a) but not detected in healthy controls. These results in agreement with earlier data in Fig. 1, confirmed that accumulation of p62 with *B. pseudomallei* infection, is due to deficiency in autophagy activation. In addition, these samples also showed predominant expression of LC3-I (18 kDa) as compared to LC3-II (16 kDa) when probed with anti-LC3 antibody (Fig. S2b). These results were found to be consistent with the p62 assay data shown in Fig. 1 and demonstrating that the increased presence of LC3-I was not associated with autophagolysomal degradation of *B. pseudomallei *, thus, linked with autophagy deficiency.

## Discussion

Our previous studies of *B. pseudomallei* infection in a murine model indicated that an increase in p62/NBR1 correlates host deficiency in autophagy activation associated with persistence of *B. pseudomallei* survival in infected mice. In this study, we examined activation of the host immune effector mechanism with a particular focus to autophagy activation in Sri Lankan melioidosis patients with confirmed *B. pseudomallei* infection. We analysed the key autophagy components p62/NBR1 in PBMCs from meliodosis patients, healthy controls in addition to *B. pseudomallei *-negative sepsis and melioidosis-negative suspected leptospirosis controls. We also measured the proinflammatory cytokine response in serum. This is the first study to our knowledge, to report an increase in p62/NBR1 levels with *B. pseudomallei* infection exhibiting a biomarker characteristic in melioidosis. Our study also hints that the increase in p62/NBR1 linked to autophagy deficiency may possibly lead to the persistence of pathogens and occurrence of recrudescence or reactivation of the disease.

In the autophagy process, unwanted cytosolic components including invasive pathogens engulfed by double-membrane-bound structures (auto-phagosomes), are delivered to lysosomes/vacuoles responsible for degradation. While *B. pseudomallei* may trigger limited autophagy by a type-three secretion system-dependent process (T3SS), T3SS probably also plays an essential part in the evasion of autophagy because (BopA) mutant bacteria are taken up more efficiently by autophagic vesicles and have decreased intracellular survival [32, 33]. Although, the complete mechanism of autophagy escape of *B. pseudomallei* is yet to be defined. Our earlier reports on a mouse model of melioidosis indicate that presence of bacterial burden correlated with increase autophagic component p62/NBR1 in acute infection and linked to autophagy deficiency. We extended this observation in human melioidosis by analysing PBMCs, from a large number of confirmed cases of melioidosis with *B. pseudomallei *, which showed a significant increase in p62/NBR1 levels. This increase in p62/NBR1 levels in confirmed cases suggest a lack of complete clearance of the pathogens because p62/NBR1 levels do not drop down to basal levels. This suggests that an increase in p62/NBR1 in acute infection fits the characteristic of a biomarker of disease. Diagnosing disease using biomarkers is dependent upon a correlation between biomarker levels and disease state, whereby biomarker levels for a certain diseased population are different usually higher than the corresponding non-diseased population. The ROC curve plot of sensitivity (Se) versus 1-speficity (Sp) at all possible concentrations provides probability of truly identifying diseased and non-diseased individuals, respectively. In this study, our results showed that the host p62/NBR1 levels' cut-off point provides a good measure, and characteristic of a biomarker.

Diagnosis of melioidosis based on clinical symptoms from a wide range of clinical mamifestations and septicemic conditions can be challenging. It requires tedious, time-consuming laboratory diagnostic tests for disease confirmation. In rural hospital settings such laboratory facilities may not be available and rapid diagnostic tests at point of care would be very useful in early diagnosis of infection, provided potential biomarkers supportive for melioidosis are identified. Non-culture-based methods to diagnose bacterial infectious diseases are increasingly being encouraged [34], but they have not yet been extensively developed and evaluated for melioidosis. In this study, our results showed that the cytosolic proteins p62 and NBR1 increased significantly in confirmed and probable cases of melioidosis exhibiting a characteristic of host biomarker in meloidosis. The increase in p62/NBR1 also correlated with LC3-I protein with no characteristic up regulation LC3-II protein, further suggesting that there is a deficiency in autophagy activation in clearing the pathogens. In this study significantly increased levels of p62/NBR1 were detected in confirmed and probable cases of melioidosis patients compared to healthy control, and the increased levels NBR1 can distinctly dicriminate *B. pseudomallei *-negative sepsis and melioidosis-negative suspected leptospirosis controls, but at present detection of increased levels of p62 can not independently discriminate between melioidosis and other bacterial infections. Our study also showed a significant increase in the level of IL-6 in confirmed melioidosis compared to TNF-α, IL-1β and IFN-γ. Increased levels of plasma IL-8 and IL-6 concentration associated with mortality, have also been reported [35]. In summary, our results suggested that the increased levels of p62/NBR1 in PBMCs with acute *B. pseudomallei* infection linked to autophagy deficiency may be used as a potential biomarker of melioidosis.

## Methods

### Reagents

The p62 and NBR1 assay kit used in this study was obtained from Enzo Life Sciences (Framingale, NY, USA). The Rabbit polyclonal p62 antibody was purchased from Cell Signaling Technology (Danvers, MA, USA). The NBR1 antibody was purchased from Thermo Fisher scientific (Grand Island, NY, USA). Anti-LC3 antibody and anti-β actin antibodies were purchased from Novus Biologicals (Centennial, CO, USA).

### Patient enrollment

Melioidosis patients older than 18 years were enrolled in this study as described elsewhere [28] . Clinical samples, matched PBMCs and sera were collected over a period of 2 years (2013–2015) from acute and confirmed cases of melioidosis during the antibiotic treatment phase. Control sera were collected from non-melioidosis sepsis patients and healthy volunteers as described previously [28]. Ethics approval was obtained from the Ethics Review Committee, Faculty of Medicine, University of Colombo, Sri Lanka; Office of Human Use and Ethics (OHU and E) of U.S. Army Medical Research Institute of Infectious Diseases (USAMRIID), and U.S. Army Medical Research and Material Command (Office of Research Protection- Human Research Protection Office (USAMRMC-ORP-HRPO).

### Confirmation of *B. pseudomallei* infections

Patients with unknown fever origin (pneumonia, sepsis and abscess) were selected for *B. pseudomallei* screening. *B. pseudomallei* from blood and other patient specimens was isolated using specialized culture techniques and real-time (RT)-PCR assay as previously described [28]. The RT-PCR probes used were the *Salmonella typhimurium lpxO* gene, targeting a homologue gene to *B. pseudomallei *, *Yersinia *-like fimbrial (YLF) and *Burkholderia thailandensis *-like flagellum and chemotaxis (BTFC) gene clusters, as previously described [13].

### Blood collection for isolating PBMCs and lysis for biochemical assays

Processing and separation of PBMCs from collected blood was described elsewhere [28]. Briefly, blood (10 ml) were drawn from patients/volunteers after informed consent of which 7 ml were collected into BD vacutainer mononuclear cell preparation tubes (catalogue no. 362761) for lymphocyte purification and 3 ml were collected into BD vacutainer EDTA tube for plasma collection. The mononuclear cells from blood were purified using standard density gradient centrifugation with Ficoll-Hypaque, and harvested from the interface. Approximately 5×10^6^ PBMCs were obtained from each sample treated with 400 µl of RIPA cell lysis buffer 2 (catalogue no. 80–1284, Enzo Life Sciences, Farmingdale, NY, USA) containing PMSF (1 mM final, catalogue no. 36 978, Thermo Fisher Scientific, IL, USA), DNase (20 µg ml^−1^ final, catalogue no. 89 836, Thermo Fisher Scientific) and protease inhibitor cocktails (5 µl, catalogue no. 1 858 566, Thermo Fisher Scientific, IL, USA). Cells with lysis buffer were incubated on ice for 30 min with occasional vortexing, then centrifuged at 10 000 ***g*** for 10 min. The aqueous phase was frozen. The cell lysates were stored at 70 °C before biochemical assays were performed.

### Cytokine analysis

Cytokines in serum were quantitatively determined using the Meso Scale Discovery (MSD) multi-spot array ultrasensitive cytokine assay kit (according to the manufacturer’s protocol) as described elsewhere [36]. Briefly, the 96-well cytokine assay plate was used. The wells were blocked with diluent 2 (according to the manufacturer’s protocol) for 30 min at room temperature. Then, 25 µl of calibrator and samples were added to the plate in triplicate for 2 h at room temperature, the plates were washed three times with 1×PBS+0.05 % Tween-20, and 25 µl of the detection antibody solution was added to each well of the plate for 2 h at room temperature with constant shaking at 400 r.p.m. Following the 2 h incubation, the plates were washed three times with 1×PBS+0.05 % Tween 20. 150 µl of 2× Read Buffer T was added to each well of the plate and analysed on the SECTOR Imager. The assay results were read using an MSD SECTOR Image 2400 incorporating a CCD. Sample cytokine concentrations were determined with Softmax Pro Version 4.6 software, using curve fit models (log-log or 4-PL) as suggested by the manufacturer of the specific cytokine.

#### Autophagy biomarkers: p62 assay

PBMC lysates were used for measuring protein concentrations. p62 assays were performed at different protein concentrations according to the manufacturer’s protocol as described elsewhere [27]. Briefly, samples and standards were added to wells coated with a monoclonal antibody to a p62-coated plate, then washed and incubated with rabbit polyclonal antibody to p62. Following incubation and washing, the enzyme (HRP)-conjugated anti-rabbit IgG was added and incubated. Finally, a colour developer substrate (TMB) was used to activate the enzyme reaction. After a 30 min incubation with TMB, stop solution was added to each well and the plate absorbance was read in a spectrophotometer at 450 nm. The amount of p62 was determined from the plot of standard curve of human p62 standards.

### NBR1 assay

PBMC lysates at various protein concentrations were used for the NBR1 assay according to the manufacturer’s protocol. Briefly, samples and standards were added to wells coated with a monoclonal antibody to NBR1. Following incubation and washing, the enzyme (HRP)-conjugated monoclonal antibody to NBR1 and a colour developer substrate (TMB) was added. After a 30 min incubation with TMB, stop solution was added to each well and the plate absorbance was read in a spectrophotometer at 450 nm. The amount of NBR1 was determined from the plot of standard curve of human NBR1 standards.

### Western-blot analysis

PBMC lysates left over after p62/NBR1 assay were used for Westernblot analysis. Samples containing 10 µg of proteins were separated by gel electrophoresis and transferred to nitrocellulose membranes as described before [27]. The membranes were blocked overnight in 1 x Tris-buffered saline (TBS) containing 0.1 % Tween-20 and 3% bovine serum albumin at room temperature. The membranes were washed extensively with 1×TBS buffer and then probed with anti-LC3 or anti-p62 polyclonal antibody followed by horseradish peroxide-conjugated secondary antibody (goat anti-rabbit). After additional rinsing with 1×TBS buffer containing 0.1 % Tween-20, the membranes were exposed to a chemiluminescent substrate in the presence of hydrogen peroxide, using Clarity Western ECLkit (BioRad). A VersaDoc Model 4000 (BioRad) imaging system was used to capture the image.

### Statistical analysis

For each analyte, the association between disease state and concentration was tested by the Kruskal–Wallis test, with post-hoc pairwise comparisons using Dunn’s test. No adjustment was applied for multiple comparisons. The ability of each analyte to discriminate the sample of healthy controls from confirmed melioidosis, was analysed by ROC curve. An approximate diagnostic cut-off was obtained by choosing the concentration yielding the maximum Youden index [37, 38]. Analysis was implemented in R statistical software version 3.1, R package dunn.test, and SAS version 9.4 Logistic procedure [39].

## Supplementary Data

Supplementary material 1
