# Supplementary material for: An increase in p62/NBR1 levels in melioidosis patients of Sri Lanka exhibit a characteristic of potential host biomarker
Source: J Med Microbiol. 2020 Aug 20;69(10):1240–8. doi: 10.1099/jmm.0.001242 (PMC7660894; doi:10.1099/jmm.0.001242)
Supplement: Supplementary material 1 [file jmm-69-1240-s001.pdf]

# An increase in p62/NBR1 levels in melioidosis patients of Sri Lanka exhibit a characteristic of potential host biomarker

Kamal U. Saikh[1*], Cyra M. Ranji[1], Robert G. Ulrich[1], Enoka Corea[2], Aruna Dharshan De Silva[3,4], and Mohan Natesan[1]

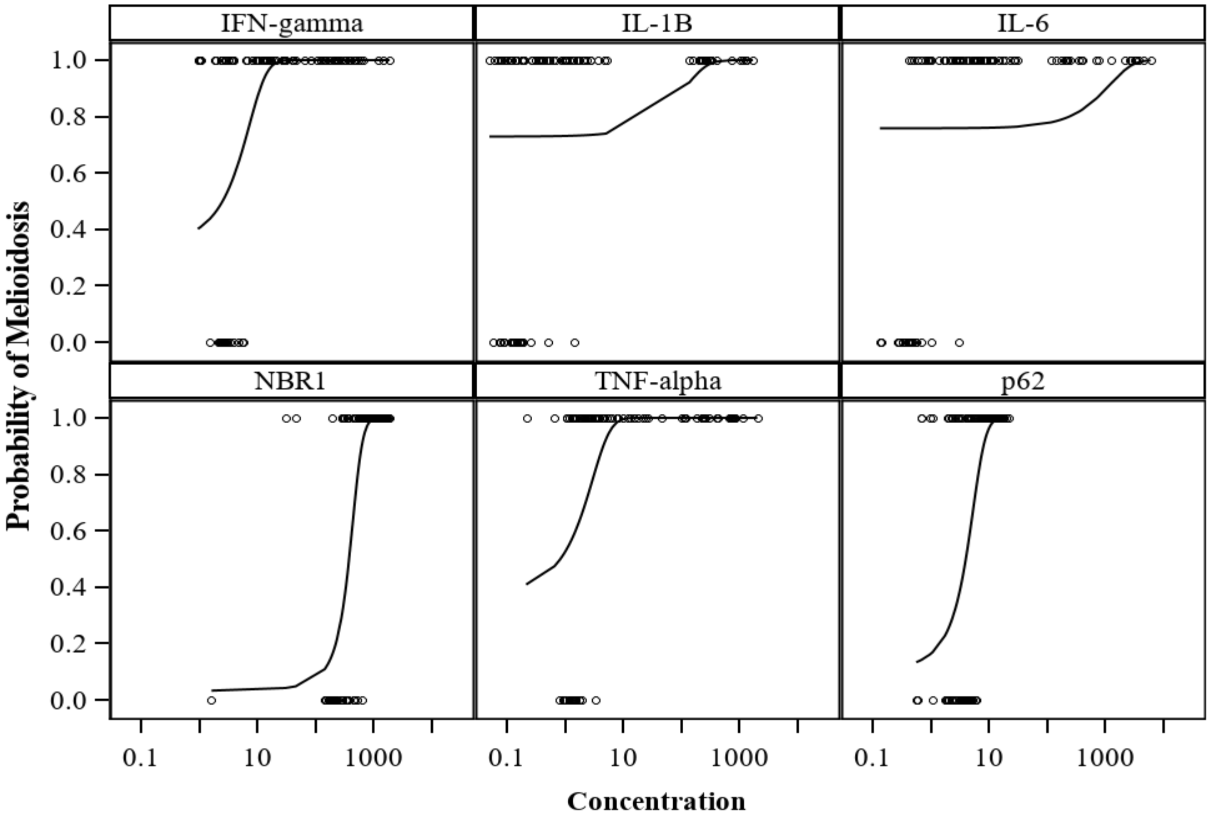

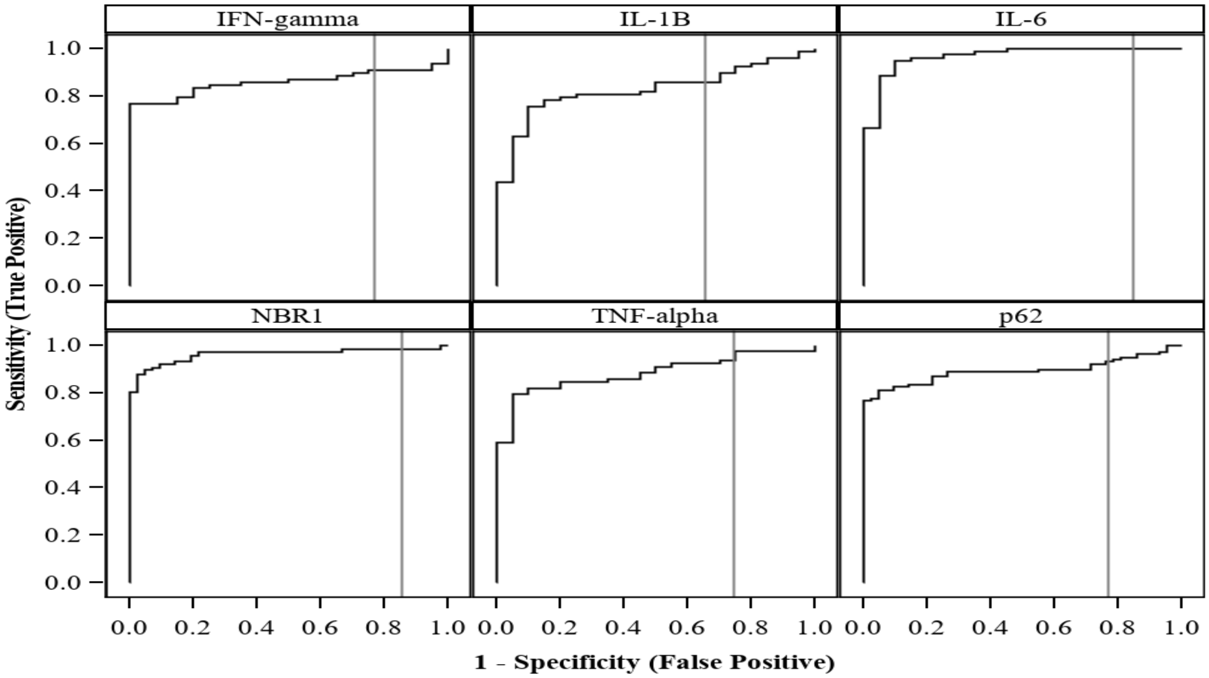


**Suppl. Fig. 1** ROC curve analysis of p62/NBR1 and pro-inflammatory cytokines for biomarker characteristics. Data analysis display p62/NBR1 and pro-inflammatory cytokines as probable and sensitive (true positive) in melioidosis samples as a characteristic of biomarker.

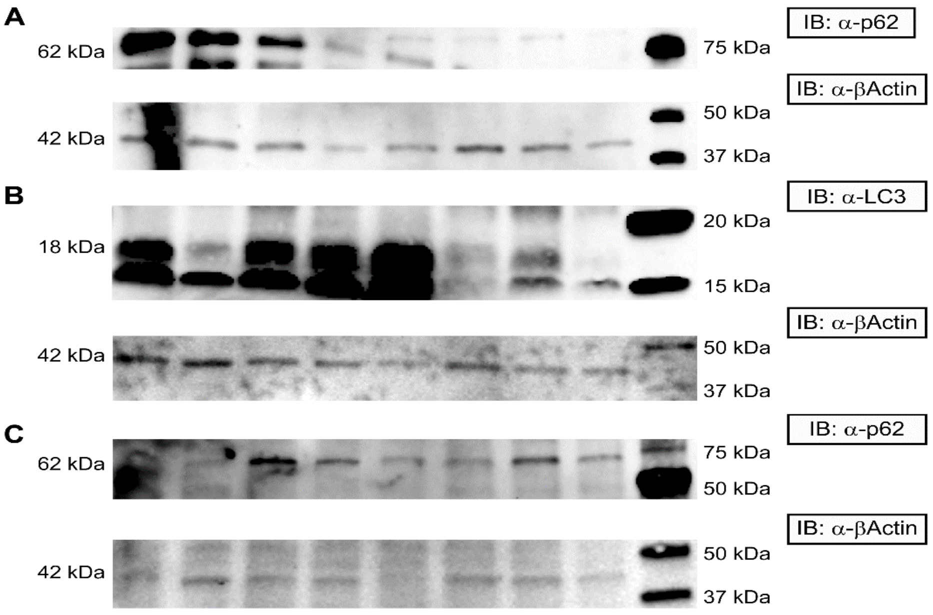

**Suppl. Fig. 2.** Intracellular accumulation of p62 and LC3 in melioidosis patients. Randomly selected samples of cell lysates of PBMCs from confirmed malioidosis, melioidosis negative and healthy controls that were available after measuring p62/NBR1by ELISA based assay were used for detecting p62 and LC3-I/LC3-II by Westerblot analysis. Samples containing 10 µg of total proteins were separated by SDS-PAGE gel electrophoresis and transferred to nitrocellulose membranes and then probed with anti-LC3 or anti-p62 polyclonal antibody followed by horseradish peroxide-conjugated secondary antibody (goat anti-rabbit). (A). Lanes 1-3 confirmed melioidosis (BRK007, BRK009, and BRK010). Lanes 4-5 suspected lepto/scrub typhus (BRK030 and BRK048). Lane 6 sepsis case (BRK055). Lanes 7-8 healthy controls (BRK087 and BRK093). (B and C) Lanes 1-3 confirmed melioidosis (BRK007, BRK031, and BRK032). Lanes 4-5 suspected lepto/scrub typhus (BRK030 and BRK048). Lane 6 sepsis case (BRK055). Lanes 7-8 healthy controls (BRK087 and BRK093).

**Supplemental Table 1. Kruskal-Wallis analysis of the interquartile range of p62/NBR1 and pro-inflammatory cytokines in confirmed and probable cases of melioidosis compared to healthy controls.**

| | | **Healthy Control** | **Confirmed** | **Convalescent** | **Neg. Sepsis** | **Neg. lepto** | **Proba ble** | **Relapsed** |
|---|---|---|---|---|---|---|---|---|
| **IFN-gamma** | n | 20 | 78 | 22 | 10 | 4 | 19 | |
| | Median | 2.64 | 27.3 | 3.94 | 9.43 | 57.43 | 18.08 | |
| | IQR | 2.34 , 3.15 | 6.52 , 196.26 | 3.12 , 5.06 | 8.55 , 11.41 | 8.14 , 115.80 | 6.96 , 132.00 | |
| | Kruskal-Wallis Test | <.0001 | | | | | | |
| | Post-hoc vs. Confirmed | <.0001 | | | | | | |
| | Post-hoc vs. Convalescent | 0.1792 | <.0001 | | | | | |
| | Post-hoc vs. Mel. neg. sepsis | 0.0241 | 0.0845 | 0.2295 | | | | |
| | Post-hoc vs. Mel.neg.sus.lepto. | 0.0065 | 0.9413 | 0.0478 | 0.2968 | | | |
| | Post-hoc vs. Probable | <.0001 | 0.7683 | 0.0021 | 0.197 | 0.8371 | | |

Post-hoc tests indicate result of Dunn test against group named in column.
No multiplicity adjustment has been applied.

| | | | | | | | | |
|---|---|---|---|---|---|---|---|---|
| **IL-1B** | n | 20 | 78 | 22 | 10 | 4 | 19 | |
| | Median | 0.14 | 1.01 | 0.13 | 0.44 | 0.14 | 1.28 | |
| | IQR | 0.10 , 0.18 | 0.27 , 200.37 | 0.05 , 0.68 | 0.31 , 0.89 | 0.09 , 0.17 | 0.38 , 261.96 | |
| | Kruskal-Wallis Test | <.0001 | | | | | | |

| | | | | | | | | |
|---|---|---|---|---|---|---|---|---|
| | Post-hoc vs. Confirmed | <.0001 | | | | | | |
| | Post-hoc vs. Convalescent | 0.7147 | <.0001 | | | | | |
| | Post-hoc vs. Mel. neg. sepsis | 0.0466 | 0.2533 | 0.0846 | | | | |
| | Post-hoc vs. Mel.neg.sus.lepto. | 0.7589 | 0.0099 | 0.6051 | 0.1125 | | | |
| | Post-hoc vs. Probable | <.0001 | 0.3329 | <.0001 | 0.1061 | 0.0043 | | |

Post-hoc tests indicate result of Dunn test against group named in column.
No multiplicity adjustment has been applied.

| | | | | | | | | |
|---|---|---|---|---|---|---|---|---|
| **IL-6** | n | 20 | 78 | 22 | 10 | 4 | 19 | |
| | Median | 0.4 | 7.55 | 1.05 | 6.34 | 0.86 | 17.34 | |
| | IQR | 0.30 , 0.50 | 2.66 , 197.62 | 0.56 , 1.47 | 5.37 , 8.35 | 0.58 , 10.79 | 4.49 , 202.17 | |
| | Kruskal-Wallis Test | <.0001 | | | | | | |
| | Post-hoc vs. Confirmed | <.0001 | | | | | | |
| | Post-hoc vs. Convalescent | 0.0771 | <.0001 | | | | | |
| | Post-hoc vs. Mel..neg. Sepsis | <.0001 | 0.6659 | 0.0064 | | | | |
| | Post-hoc vs. Mel neg.sus, lepto | 0.1779 | 0.0526 | 0.7242 | 0.1515 | | | |
| | Post-hoc vs. Probable | <.0001 | 0.6558 | <.0001 | 0.5073 | 0.0441 | | |

Post-hoc tests indicate result of Dunn test against group named in column.
No multiplicity adjustment has been applied.

| | | | | | | | | |
|---|---|---|---|---|---|---|---|---|
| **NBR1** | n | 42 | 116 | 27 | 28 | 34 | 46 | 2 |
| | Median | 213.14 | 1196.06 | 220.83 | 232.23 | 236.9 | 823.76 | 730.69 |
| | IQR | 180.23. 284.36 | 695.54 , 1449.66 | 142.43 , 459.70 | 168.59 , 277.54 | 199.98 , 468.25 | 399.64 , 918.50 | 198.05 ,1263 |
| | Kruskal-Wallis Test | <.0001 | | | | | | |
| | Post-hoc vs. Confirmed | <.0001 | | | | | | |
| | Post-hoc vs. Convalescent | 0.5774 | <.0001 | | | | | |
| | Post-hoc vs. Mel. neg. Sepsis | 0.9831 | <.0001 | 0.6238 | | | | |
| | Post-hoc vs. Me..neg.sus.lepto | 0.3153 | <.0001 | 0.7147 | 0.3748 | | | |
| | Post-hoc vs. Probable | <.0001 | 0.0057 | <.0001 | <.0001 | <.0001 | | |
| | Post-hoc vs. Relapsed | 0.2228 | 0.2438 | 0.3094 | 0.2308 | 0.3712 | 0.6288 | |

Post-hoc tests indicate result of Dunn test against group named in column.
No multiplicity adjustment has been applied.

| | | | | | | | | |
|---|---|---|---|---|---|---|---|---|
| **TNF-alpha** | n | 20 | 78 | 22 | 10 | 4 | 19 | |
| | Median | 1.36 | 4.53 | 2.76 | 6.94 | 1.91 | 6.11 | |
| | IQR | 1.10 , 1.49 | 2.10 , 115.64 | 1.30 , 3.34 | 3.73 , 8.53 | 1.22 , 8.37 | 2.89 , 65.11 | |
| | Kruskal-Wallis Test | <.0001 | | | | | | |
| | Post-hoc vs. Confirmed | <.0001 | | | | | | |
| | Post-hoc vs. Convalescent | 0.0626 | 0.0011 | | | | | |
| | Post-hoc vs. Mel. neg. Sepsis | <.0001 | 0.5539 | 0.0095 | | | | |
| | Post-hoc vs. Mel. neg.sus. lepto | 0.3049 | 0.1172 | 0.9804 | 0.0903 | | | |
| | Post-hoc vs. Probable | <.0001 | 0.6168 | 0.0034 | 0.8562 | 0.0905 | | |

Post-hoc tests indicate result of Dunn test against group named in column.
No multiplicity adjustment has been applied.

| | | | | | | | | |
|---|---|---|---|---|---|---|---|---|
| **p62** | n | 42 | 116 | 27 | 28 | 34 | 46 | 2 |
| | Median | 3.49 | 10.88 | 2.89 | 4.16 | 6.97 | 8.26 | 3.65 |
| | IQR | 2.68 , 4.11 | 6.75 , 12.86 | 1.09 , 4.25 | 2.99 , 6.42 | 5.70 , 9.58 | 6.70 , 10.27 | 1.42 , 5.88 |
| | Kruskal-Wallis Test | <.0001 | | | | | | |
| | Post-hoc vs. Confirmed | <.0001 | | | | | | |
| | Post-hoc vs. Convalescent | 0.9615 | <.0001 | | | | | |
| | Post-hoc vs. Mel. neg. sepsis | 0.0582 | <.0001 | 0.0951 | | | | |
| | Post-hoc vs. Mel. neg.sus.lepto | <.0001 | 0.0061 | 0.0002 | 0.0412 | | | |
| | Post-hoc vs. Probable | <.0001 | 0.2288 | <.0001 | 0.0004 | 0.1501 | | |

| | | | | | | |
|---|---|---|---|---|---|---|
| Post-hoc vs. Relapsed | 0.8853 | 0.0474 | 0.8996 | 0.625 | 0.2271 | 0.0954 |

Post-hoc tests indicate result of Dunn test against group named in column.
No multiplicity adjustment has been applied.
